# Disturbance of DNA conformation by the binding of testosterone-based platinum drugs via groove-face and intercalative interactions: a molecular dynamics simulation study

**DOI:** 10.1186/1472-6807-13-4

**Published:** 2013-03-22

**Authors:** Shanshan Cui, Yan Wang, Guangju Chen

**Affiliations:** 1 Key Laboratory of Theoretical and Computational Photochemistry of Ministry of Education, College of Chemistry, Beijing Normal University, Beijing 100875, P. R. China; 2 Present address: College of Chemistry, Beijing Normal University, 19# Xinjiekouwai Street, Haidian District, Beijing 100875, PR China

**Keywords:** Molecular dynamics simulations, Groove-face and intercalative interactions, Testosterone-based platinum agent, Pt + DNA adducts, DNA conformation distortion

## Abstract

**Background:**

To explore novel platinum-based anticancer agents that are distinct from the structure and interaction mode of the traditional cisplatin by forming the bifunctional intrastrand 1,2 GpG adduct, the monofunctional platinum + DNA adducts with extensive non-covalent interactions had been studied. It was reported that the monofunctional testosterone-based platinum(II) agents present the high anticancer activity. Moreover, it was also found that the testosterone-based platinum agents could cause the DNA helix to undergo significant unwinding and bending over the non-testosterone-based platinum agents. However, the interaction mechanisms of these platinum agents with DNA at the atomic level are not yet clear so far.

**Results:**

In the present work, we used molecular dynamics (MD) simulations and DNA conformational dynamics calculations to study the DNA distortion properties of the testosterone-based platinum + DNA, the improved testosterone-based platinum + DNA and the non-testosterone-based platinum + DNA adducts. The results show that the intercalative interaction of the improved flexible testosterone-based platinum agent with DNA molecule could cause larger DNA conformational distortion than the groove-face interaction of the rigid testosterone-based platinum agent with DNA molecule. Further investigations for the non-testosterone-based platinum agent reveal the occurrence of insignificant change of DNA conformation due to the absence of testosterone ligand in such agent. Based on the DNA dynamics analysis, the DNA base motions relating to DNA groove parameter changes and hydrogen bond destruction of DNA base pairs were also discussed in this work.

**Conclusions:**

The flexible linker in the improved testosterone-based platinum agent causes an intercalative interaction with DNA in the improved testosterone-based platinum + DNA adduct, which is different from the groove-face interaction caused by a rigid linker in the testosterone-based platinum agent. The present investigations provide useful information of DNA conformation affected by a testosterone-based platinum complex at the atomic level.

## Background

Since the discovery of cisplatin [*cis*-(NH_3_)_2_PtCl_2_], as an anticancer agent, a series of platinum anticancer drugs were used in treatment of various cancers in clinical chemotherapy [1-6]. The platinum(II) center of traditional cisplatin reacts with DNA forming two covalent bonds to N7 atoms of two adjacent guanine (G) bases with the bifunctional intrastrand 1,2 GpG adduct, to prevent the replication and transcription of cancer genes, and ultimately to induce tumor cell apoptosis [6-9]. However, the efficacy of traditional cisplatin is often compromised because of the intrinsic and acquired resistance, as well as the toxic side effects [3,10-14]. Much effort has been devoted to the developments of novel platinum-based anticancer agents which might form monofunctional platinum + DNA adducts with extensive non-covalent interactions to circumvent such drawbacks [11,15,16]. The structures of these monofunctional platinum + DNA adducts with extensive non-covalent interactions are different from that produced by cisplatin [8,9]. To the best of our knowledge, the non-covalent interactions in platinum + DNA adducts, mostly resulting from the interactions between the ligands of platinum agents and DNA molecules, include the electrostatic interaction, groove-face interaction and intercalative interaction [17-22]. Especially, the groove-face and intercalative interactions could greatly stabilize the distorted DNA molecule via hydrogen bonds and hydrophobic interactions, etc [17,18,20,21]. Therefore, some pioneering strategies toward improving the ligand properties of monofunctional platinum agents have emerged [15,23-27]. However, the systematic studies on the relationship between the ligand properties of platinum agents and interaction modes in the platinum + DNA adducts have not yet been clearly detailed so far.

Recently, it has been shown that the monofunctional platinum complexes of nitrogen-containing heterocyclic amines, such as pyridine, pyrimidine, purine, piperidine, picoline, and their derivatives could enhance cytotoxicity, perhaps because of formations of monofunctional platinum + DNA adducts rather than those of bifunctional ones [15,23]. Especially, Hannon and co-workers reported that the linkage of a testosterone to aromatic *N*-heteroatomic monofunctional platinum(II) agents confers relatively high activity to otherwise non-active platinum(II) agents. Moreover, they indicated that the conjugation of testosterone enhances the delivery ability into the tumor cell, and inherent antitumor activity of testosterone-based platinum agents [26]. Importantly, it was also proved that the testosterone-based platinum agents cause the DNA helix to undergo significant unwinding and bending over the non-testosterone-based platinum agents. This might be caused by the steric bulk of testosterone which requires greater unwinding/bending of DNA helix to accommodate the agent in the DNA double helix [27]. Theoretically, the structures of some new designed drugs were usually optimized by the density functional theory (DFT) method [28]. Some molecular dynamics (MD) simulations were used for investigating the interaction properties between DNA molecule and different platinum agents, such as cisplatin, oxaliplatin, [PtCl(en)(ACRAMTU-*S*)](NO_3_)_2_ and so on [29-36]. Moreover, the MD simulations were also used for investigating the molecular interactions between the androgen receptor protein and testosterones in the platinum agents [24,37]. However, few theoretical studies devoted to the interactions between the monofunctional *N*-heteroatomic platinum agents with the testosterone ligands and DNA molecules.

Delineating the structural details of DNA bound by platinum agents will help us to understand the features that are responsible for the remarkable potency of these platinum agents. Nevertheless, how the testosterone ligands affect the interaction modes of platinum agents with DNA molecules is still unknown. In the present work, we used molecular dynamics simulations and DNA dynamics analysis to study the conformational properties of DNA disturbed by different monofunctional *N*-heteroatomic platinum agents. Due to the potent antitumor activity of *cis*-[Pt(Testo)Cl][NO_3_] ( Testo = (NH_3_)_2_(17α-pyridyl-3-ethynyltestosterone)) as a rigid testosterone-based platinum agent studied by Hannon and co-workers [26], the first MD simulation was performed on the Pt(Testo)(II) + DNA adduct to investigate interaction properties of this platinum agent with DNA, and effects of the testosterone ligand on DNA conformation. To better understand the effects of the testosterone ligand on interaction modes in the platinum + DNA adducts, the second MD simulation was performed on the improved Pt(Testo)(II) agent with flexible linker interacting with DNA molecule. To compare with these two testosterone-based platinum agents, a non-testosterone-based platinum agent adding to DNA molecule has also been simulated.

## Methods

### Initial structures

**Figure 1:**
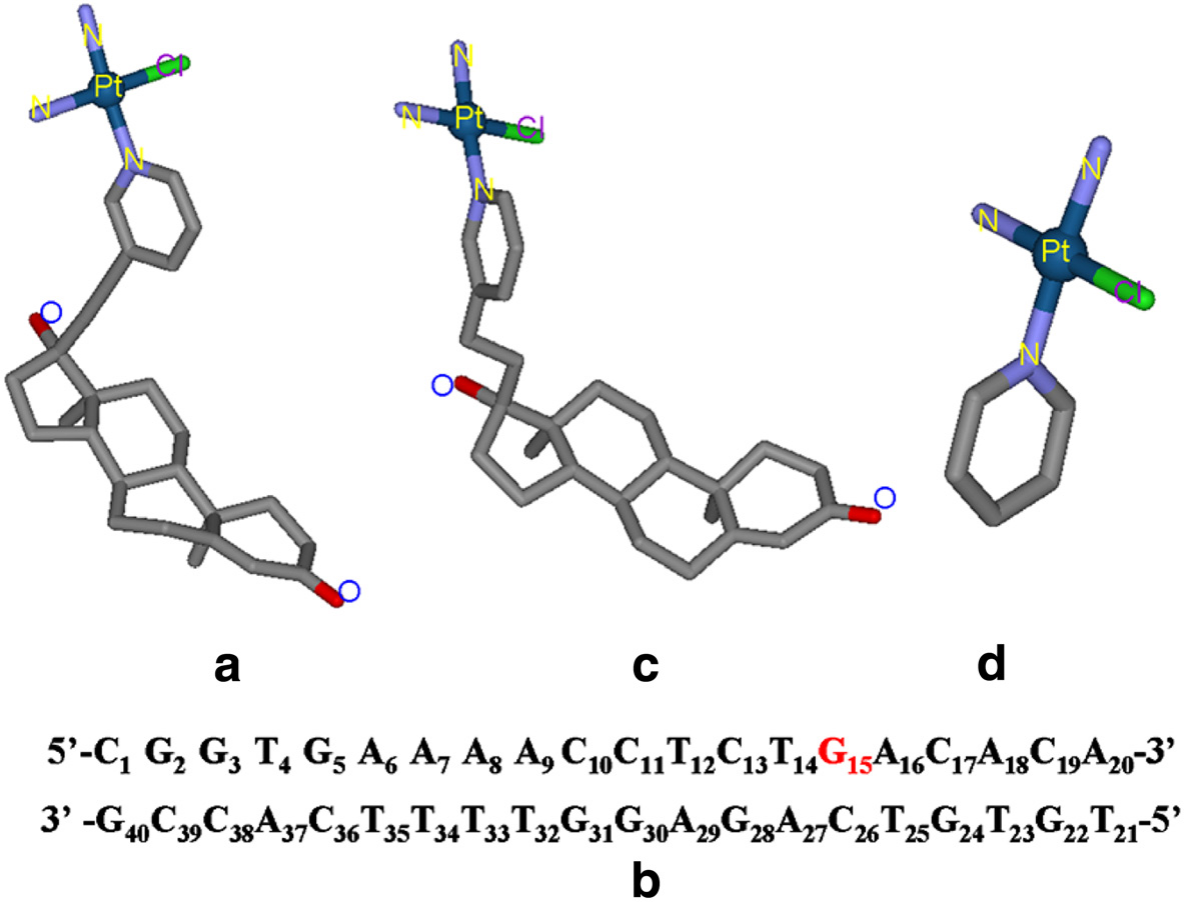
**Calculated structures of the studied platinum agents and the DNA sequence.** Calculated structures of the studied platinum agents: (**a**) Pt(Testo)(II), (**b**) the 20bp DNA sequence ( The G15 base is the binding site to platinum center), (**c**) Im-Pt(Testo)(II), (**d**) Pt(Py)(II).

On the basis of previous experimental studies [26,27], we choose the initial structure of the *cis*-[ Pt(Testo)Cl][NO_3_] ( Testo = (NH_3_)_2_(17α-pyridyl-3-ethynyltestosterone)) from the geometry optimized by the DFT method at the B3LYP/6-31G** + LanL2DZ level of theory due to the inexistence of its crystal structure (see Figure 1(a)). The corresponding geometry parameters with the available experimental data from similar molecules are shown in Additional file 1: Table S1 and Additional file 2: Figure S1. The 20bp DNA fragment, 5^′^-d(CGGTGAAAACCTCTGACACA)-3^′^, was chosen from the primer PUC19 F studied by the experiment of polymerase-chain reaction (PCR) [27] and shown in Figure 1(b). To build a Pt(Testo)(II) + DNA adduct, its initial coordinates used in our simulations were generated by manually docking [38-40]. Namely, the platinum center is bound to the N7 atom of G15 base in the major groove of DNA due to the experimental prediction for a similar platinum agent [25]. The plane of platinum center coordinated by four ligands is perpendicular to the plane of G15 base; ultimately, the linked pyridyl ring is approximately perpendicular to the base pair planes of DNA, and is parallel to the wall of major groove of DNA; the testosterone ligand with four 5/6-membered rings linked to the pyridyl ring is approximately localized around the T14:A27 base pair across the major groove of DNA, minimising the steric overlap between the agent and the DNA molecule. Since several different orientations of platinum center plane to the G15 base of DNA for the Pt(Testo)(II) + DNA adduct were chosen as the starting structures for our MD simulations, this provides a good test of whether our MD simulations are capable of driving significantly distinct starting structures to a non-distinguishable one when simulations reach equilibrium. To further investigate the influence of testosterone ligand of platinum agent on interaction modes, we improved the flexibility of linker between the pyridyl and testosterone ligands in the Pt(Testo)(II) agent with changing the linker of ethynyl –C ≡ C– to that of ethyl –CH_2_–CH_2_– (assigned as Im-Pt(Testo)(II) agent); simultaneously a non-testosterone-based platinum agent, *cis*-[Pt(NH_3_)_2_(pyridine)Cl][NO_3_] (assigned as Pt(Py)(II) agent), was also chosen to perform a comparison. The Im-Pt(Testo)(II) and Pt(Py)(II) agents were fully optimized by the same method used for the Pt(Testo)(II) agent, and shown in Figure 1(c) and (d) [26,27]. The Im-Pt(Testo)(II) + DNA and Pt(Py) (II) + DNA adducts were similarly built by using the same method for the Pt(Testo)(II) + DNA adduct described above. To compare the differences between the DNA adduct conformations and an undamaged DNA molecule, a bare-DNA (B-DNA) molecule simulation was also performed, in which an idealized B-DNA with the same base-pair sequence in these adducts was used as a starting structure for the simulation. Given that each strand of DNA has some phosphate groups, Na^+^ counterions were added to each system to achieve electroneutrality. The systems were explicitly solvated using the TIP3P water potential inside a box large enough to ensure the solvent shell extended to 10 Å in all directions.

### Force field parameter preparation

The atom types for the studied platinum agents, except for the platinum atoms, were generated using the ANTECHAMBER module in the AMBER9 program [41]. The electrostatic potentials of the platinum agents used for RESP charge calculations were calculated at the B3LYP/6-31G** + LanL2DZ [28,31,42,43] level of theory using the Gaussian09 program [44]. The RESP charges of platinum agents were derived by the RESP program based on the calculated electrostatic potentials. The force field parameters around the Pt center were generated by quantum chemical calculations, which was reported particularly in our previous work [45,46]. Other force field parameters of the platinum agents were generated from the gaff force field in the AMBER9 program.

### Molecular dynamics simulations protocols

All MD simulations were carried out using the AMBER9 package [41] with the parm99 force field [47,48], the parmbsc0 refinement [49] and gaff [50] force field parameters. The details of MD protocols are given in Additional file 3: Methodology.

### Principal component analysis

Principal component analysis can be used to segregate large-scale correlated motions from random thermal fluctuations, thereby probing the essential dynamics of the system. The details of this analysis method are available in Additional file 3: Methodology.

### DNA groove parameter analyses

The frequency distributions (fraction of the time spent in each conformation) from the trajectories of simulations for the models and a canonical B-DNA were calculated using the CURVES program [51] to investigate the distortion of DNA. To account for the distortion of whole DNA backbone, the overall bend, tilt and roll angles of the DNA time-averaged structures for the studied models were calculated by using the MadBend program from the CURVES outputs [52]. The details of the calculation method are available in Additional file 3: Methodology.

## Results

**Figure 2:**
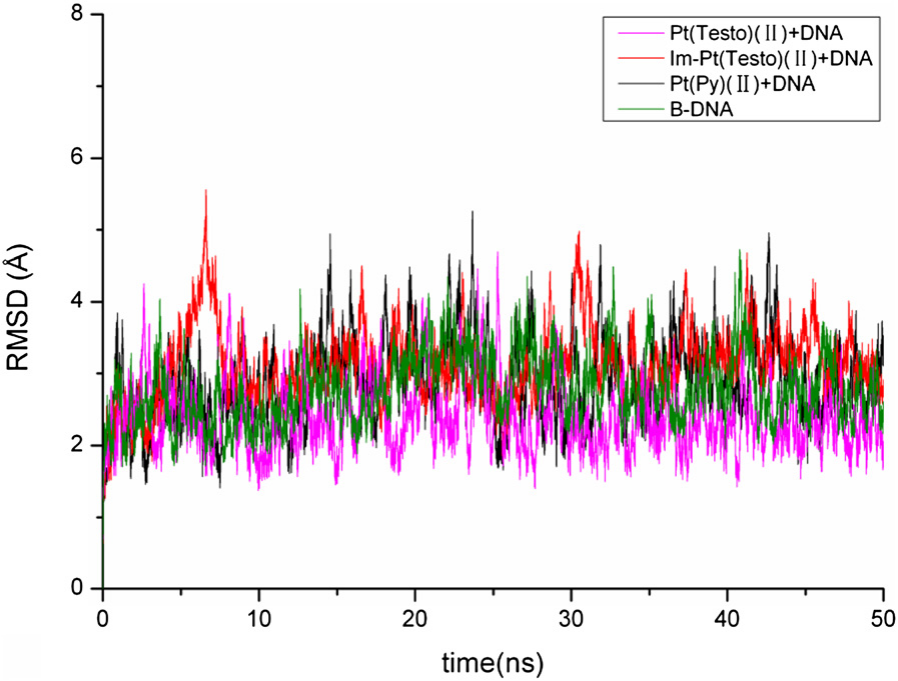
**RMSD values of Pt(Testo)(II) + DNA, Im-Pt(Testo)(II) + DNA, Pt(Py)(II) + DNA and B-DNA models.** RMSD values of all backbone atoms in the simulations of Pt(Testo)(II) + DNA (magenta), Im-Pt(Testo)(II) + DNA (red), Pt(Py)(II) + DNA (black) and B-DNA (olive) models with respect to the corresponding starting structure.

The root-mean-square deviation (RMSD) values of all backbone atoms referenced to the corresponding starting structures over all three trajectories for the Pt(Testo)(II) + DNA, Im-Pt(Testo)(II) + DNA and Pt(Py)(II) + DNA models were examined to determine if each system had attained equilibrium. It is often considered that small RMSD values of a simulation indicate a stable state of the system. Plots of RMSDs of three system simulations over times are shown in Figure 2. It can be seen from Figure 2 that each platinum + DNA adduct reached equilibrium after 30 ns, and their energies were found to be stable during the remainder of each simulation. Therefore, the trajectory analysis for each of three systems has extracted the equilibrated conformation between 30 ns and 50 ns of simulation time, recording 10000 snapshots at every 2 ps time-interval of each trajectory.

### Disturbance of DNA conformation by the binding of Pt(Testo)(II) agent via groove-face interaction

#### Conformation analysis of Pt(Testo)(II) + DNA adduct

**Figure 3:**
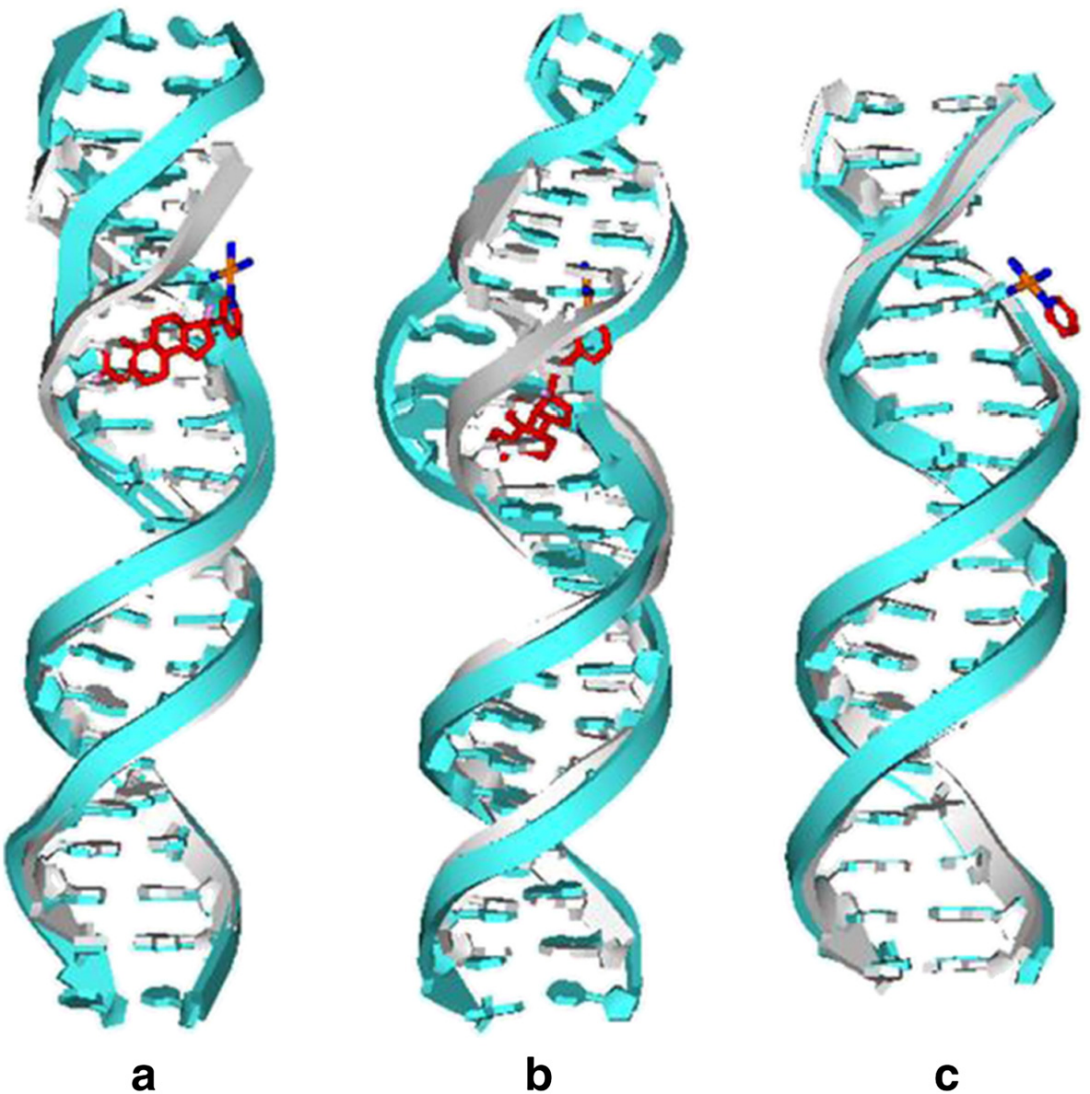
**The average centroid structures of three adducts with an undamaged B-DNA.** The average centroid structures of three adducts : (**a**) Pt(Testo)(II) + DNA, (**b**) Im-Pt(Testo)(II) + DNA and (**c**) Pt(Py)(II) + DNA (the double-strand DNA backbones (cyan) and the studied platinum agents in stick (Pt: orange, N: blue, C: red, O: pink)) along with an undamaged B-DNA (light gray).

Based on the results of previous experiment [27], the testosterone-based platinum agent, *cis*-[Pt(NH_3_)_2_(17α-pyridyl-3-ethynyltestosterone)Cl]^+^ (called Pt(Testo)(II)), which consists of testosterone, ethynyl linker and platinum(II) center coordinated by one chlorine atom and three nitrogen atoms from two ammonias and one pyridyl, as an efficient anticancer agent, binding to a DNA molecule (called Pt(Testo)(II) + DNA adduct), was tested by MD simulation. The average structure of Pt(Testo)(II) + DNA adduct was obtained by the trajectory analysis extracting the equilibrated conformations between 30 ns and 50 ns of simulation times and shown in Figure 3 along with that of undamaged DNA molecule. It is demonstrated from this average structure that the testosterone with a groove-face interaction mode locates at the major groove of DNA molecule; the platinum center binds to the N7 atom of G15 base of DNA molecule; the pyridyl ring is perpendicular to the plane of G15 base of DNA; the plane of testosterone with the rigid ethynyl (–C ≡ C–) linker is perpendicular to the pyridyl ring and plane of T14:A27 base pair of DNA molecule.

**Figure 4:**
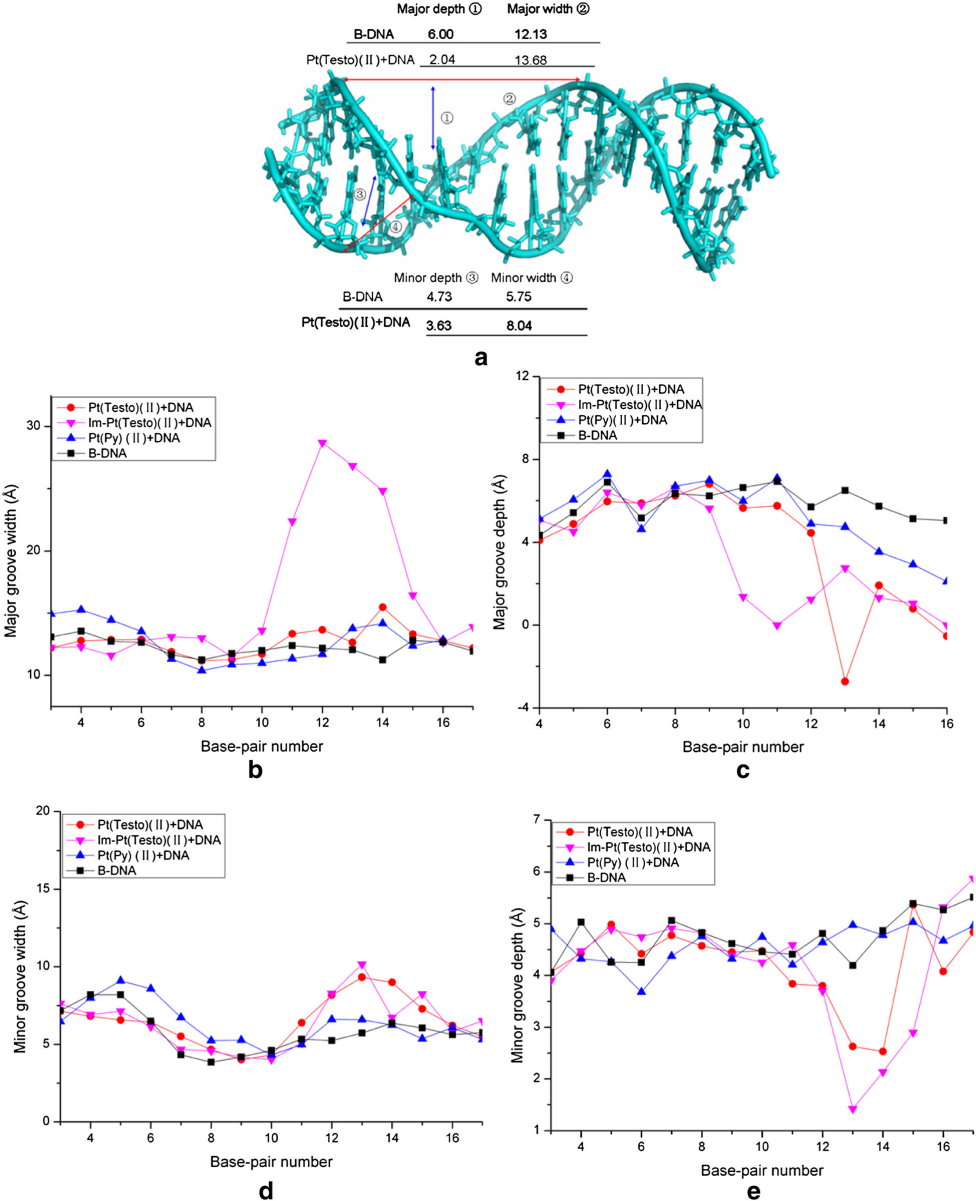
**Groove widths and depths of B-DNA, Pt(Testo)(II) + DNA, Im-Pt(Testo)(II) + DNA and Pt(Py)(II) + DNA models.** (**a**) The scheme of the minor and major groove widths/depths for the time-average structures of DNA conformations for the Pt(Testo)(II) + DNA adduct and B-DNA. (**b**) The major groove width; (**c**) the major groove depth; (**d**) the minor groove width; (**e**) the minor groove depth for the time-average structures of DNA conformations for the Pt(Testo)(II) + DNA adduct (red lines with circles), Im-Pt(Testo)(II) + DNA adduct (Magenta lines with down-triangles), Pt(Py)(II) + DNA adduct (blue lines with up-triangles) and B-DNA (black lines with square symbols) (black lines with squares).

Because the testosterone ligand locates at the DNA major groove via the groove-face interaction mode, both major groove and minor groove of DNA molecule tend to widen and shoal. Figure 4 shows the DNA groove parameters around the connection sites, C11:G30 ~ G15:C26 base pairs of DNA, which are near to the testosterone position, for the Pt(Testo)(II) + DNA and B-DNA models. Namely, the average major groove width and minor groove width increase from 12.13 to 13.68 Å and from 5.75 to 8.04 Å, respectively, compared to a normal B-DNA; then their depths are shoaled from 6.00 to 2.04 Å and from 4.73 to 3.63 Å, respectively (Figure 4(a), (b), (c), (d) and (e); see the red lines with circles and the black lines with squares for the Pt(Testo)(II) + DNA and B-DNA models, respectively). In addition, the measured degrees of overall DNA bend, tilt and roll angles for the bound-DNA backbone conformation of Pt(Testo)(II) + DNA adduct are shown in Table 1. It can be seen that the average deviation percentage of helical angles with respect to the undamaged B-DNA molecule is 261.64% (260.02% for bend; 270.54% for tilt; 254.37% for roll) for the Pt(Testo)(II) + DNA adduct. Interestingly, the bend angle toward the major groove was observed, which suggests there is great stability of this adduct for this orientation with the groove-face interaction mode. As expected, the testosterone-based platinum agent could cause a great DNA helical conformation distortion, which is consistent with the previous experimental study [27].

**Table 1:** Values of average overall bend, tilt and roll angles (°) for the DNA conformations of the studied adducts and undamaged B-DNA

**Adducts**	**Bend**	**Tilt**	**Roll**
B-DNA	11.73	6.89	9.49
Pt(Testo)(II) + DNA	42.23	25.53	33.63
Im-Pt(Testo)(II) + DNA	47.46	45.03	14.96
Pt(Py)(II) + DNA	28.49	17.19	22.72

#### Hydrogen bond and hydrophobic interaction analyses of Pt(Testo)(II) + DNA adduct

**Figure 5:**
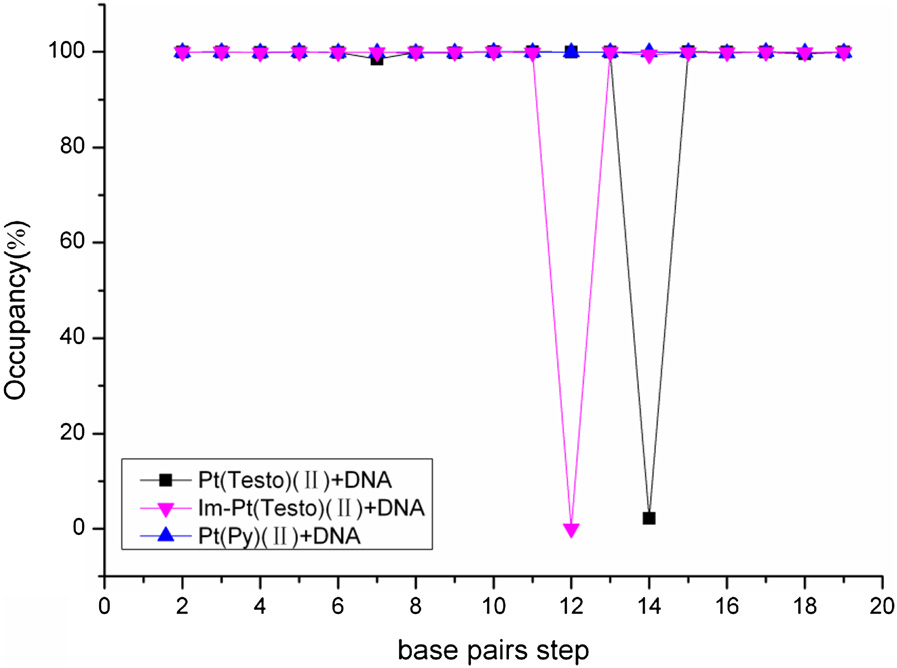
**Hydrogen bond occupancies of base pairs along with base-pair steps for three adducts.** Hydrogen bond occupancies of base pairs along with base-pair steps for each adduct: Pt(Testo)(II) + DNA (black lines with squares); Im-Pt(Testo)(II) + DNA (Magenta lines with down-triangles); Pt(Py)(II) + DNA (blue lines with up-triangles).

**Figure 6:**
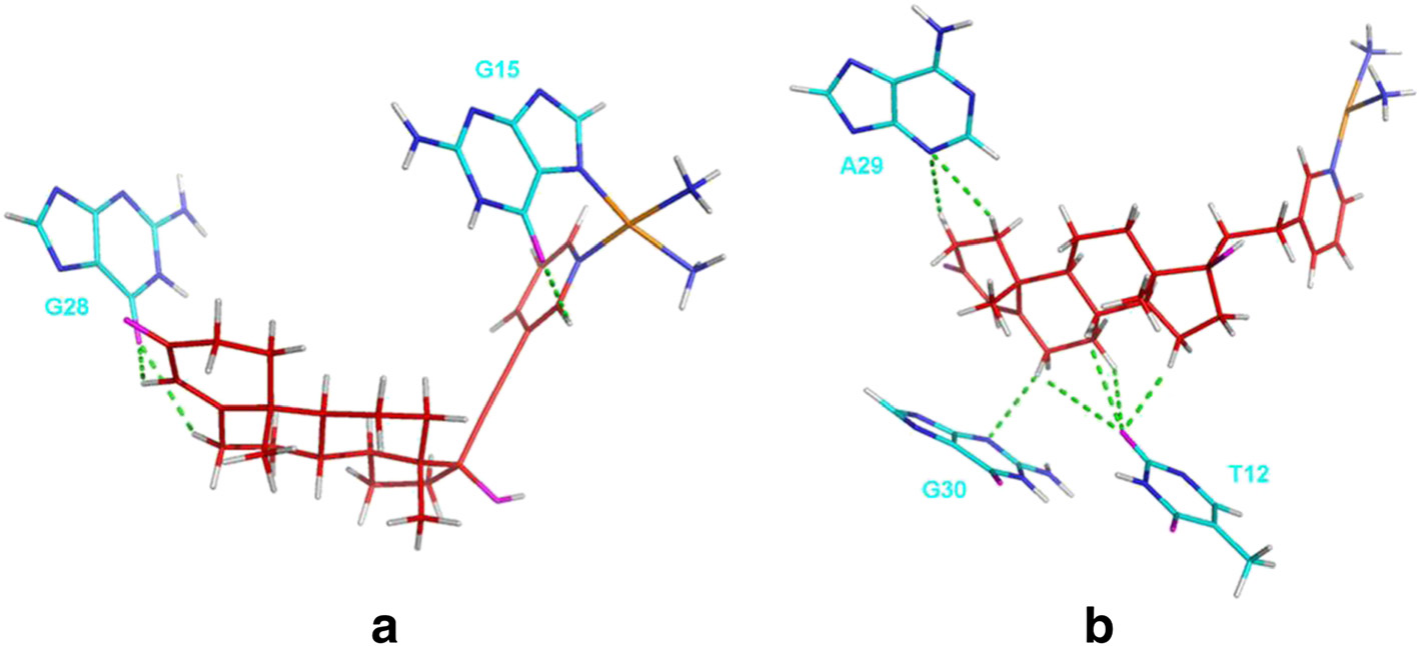
**Hydrogen bonds between platinum ligands and DNA molecule.** Hydrogen bonds between platinum ligands and DNA molecule: (**a**) Pt(Testo)(II) + DNA and (**b**) Im-Pt(Testo)(II) + DNA (the DNA bases and the platinum complexes in tube, platinum atoms in orange, nitrogen atoms in blue, oxygen atoms in Magenta, hydrogen atoms in white, carbon atoms of DNA molecule in cyan and carbon atoms of the complexes in red).

To address the extent of DNA distortion through the groove-face interaction mode, the destruction of hydrogen bonds in the DNA molecule and the formation of new hydrogen bonds between the Pt agent and DNA molecule have been analyzed and shown in Figure 5, Figure 6(a) and Table 2, respectively. A distance of less than 3.5 Å and an angle of greater than 120° between the potential hydrogen bond donor and acceptor were used as the criteria for a hydrogen bond formation [33,53]. Due to the interaction of testosterone ligand with DNA bases at the major groove, the hydrogen bonds at T14:A27 base pair in DNA molecule are completely destroyed compared with the undamaged B-DNA (see Figure 5). The destructions of hydrogen bonds result from the formations of some new hydrogen bonds between the C-H groups of testosterone ligand and O6 atom of G28 base, and between the C-H groups of pyridyl ligand and O6 atom of G15 base with 20.82% and 13.78% occupancy times, respectively (see Table 2 and Figure 6(a)). Moreover, hydrophobic interactions, defined as a distance between carbon atoms shorter than 4.5 Å, have been analyzed and shown in Table 3. Namely, it was found that the hydrophobic interactions between the carbon atoms of testosterone ligand and carbon atoms of C26, A27 and T14 bases of DNA occur with the occupancy times of 211.69%, 36.08% and 23.44%, respectively. Both hydrogen bond destruction in DNA molecule and new interaction formation of Pt(Testo)(II)-DNA interface facilitate the DNA distortion, which might make DNA repair more difficult.

**Table 2:** Occupancies of hydrogen bonds between carbon hydrogen atoms of platinum agent (electron acceptor) and near bases on DNA (electron donor)

**Adducts**	**Electron donor**	**Electron acceptor**	**Occupancy (%)**
Pt(Testo)(II) + DNA	G28/O6	H-C_testosterone_	20.82
	G15/O6	H-C_pyridyl_	13.78
Im-Pt(Testo)(II) + DNA	T12/O2	H-C_testosterone_	58.57
	A29/N3	H-C_testosterone_	8.92
	G30/N3	H-C_testosterone_	5.25

**Table 3:** Occupancies of hydrophobic interactions between carbon atoms of platinum agent and near bases on DNA

**Adducts**	**Ligand**	**DNA**	**Occupancy (%)**
Pt(Testo)(II) + DNA	C_testosterone_	C26	211.69
	C_testosterone_	A27	36.08
	C_testosterone_	T14	23.44
Im-Pt(Testo)(II) + DNA	C_testosterone_	T12	198.67
	C_testosterone_	C13	159.99
	C_testosterone_	A29	41.26
	C_testosterone_	G30	40.67
	C_ethyl_	T14	9.12

### Disturbance of DNA conformation by the binding of Im-Pt(Testo)(II) agent via intercalative interaction

It has been shown from the structure analysis of Pt(Testo)(II) + DNA adduct discussed above that the Pt(Testo)(II) agent interacts with DNA molecule adopting the groove-face interaction mode due to the rigid ethynyl –C ≡ C– linker between the testosterone and pyridyl ligands. To produce great effects of Pt(Testo)(II) agent on DNA conformation distortion via intercalative interaction mode, the rigid ethynyl –C ≡ C– linker in the Pt(Testo)(II) agent was changed to a flexible linker, ethyl –CH_2_–CH_2_–, in order to build a new modified platinum agent, called Im-Pt(Testo)(II). The Im-Pt(Testo)(II) agent, as an improved anticancer agent, binding to a DNA molecule (called Im-Pt(Testo)(II) + DNA adduct) was also tested by MD simulation. The average structure of Im-Pt(Testo)(II) + DNA adduct are shown in Figure 3 along with that of undamaged DNA molecule, and demonstrates that the plane of testosterone ligand embeds into the middle of T12 and C13 bases of DNA molecule at the DNA major groove via an intercalative interaction mode, and parallels to both T12 and C13 bases; the platinum center also binds to the N7 atom of G15 base of DNA molecule; the pyridyl ring is still perpendicular to the plane of G15 base of DNA. The DNA conformation distortion greatly increases due to the modified platinum agent interacting with DNA molecule via the intercalative interaction mode.

Because the testosterone ligand embeds into the bases of DNA molecule at the major groove via the intercalative interaction mode, the major and minor grooves of DNA molecule in the Im-Pt(Testo)(II) + DNA adduct become wider and shallower than those in the Pt(Testo)(II) + DNA adduct. Namely, the average width of DNA major groove in the Im-Pt(Testo)(II) + DNA adduct increases by 74.27% (from 13.68 Å to 23.84 Å) compared to the Pt(Testo)(II) + DNA adduct; then the average major groove depth and minor groove depth are shoaled by 14.71% (from 2.04 Å to 1.74 Å) and by 18.73% (from 3.63 Å to 2.95 Å), respectively (Figure 4(b), (c) and (e); see the red lines with circles and the Magenta lines with down-triangles for Pt(Testo)(II) + DNA and Im-Pt(Testo)(II) + DNA adducts, respectively). In addition, the average deviation percentage of helical angles for the Im-Pt(Testo)(II) + DNA adduct is larger by 43.63% than that for the Pt(Testo)(II) + DNA adduct (see Table 1). Similarly, the occupancy percentages of formations of new hydrogen bonds and hydrophobic interactions in the Im-Pt(Testo)(II) + DNA adduct are larger than those in the Pt(Testo)(II) + DNA adduct due to the intercalative interaction mode, except for the same extent destruction of hydrogen bonds at the T12:A29 base pair of DNA molecule compared with the Pt(Testo)(II) + DNA adduct. Namely, the Im-Pt(Testo)(II) + DNA adduct spends more occupancy times by 38.14% (from 34.60% to 72.74%) forming new hydrogen bonds between the C-H groups of testosterone ligand and the O2/N3/N3 atom of T12/A29/G30 base than the Pt(Testo)(II) + DNA adduct (shown in Figure 6(b)); simultaneously, it still spends more occupancy times by 178.5% (from 271.21% to 449.71%) forming more hydrophobic interactions between C atoms of testosterone and C atoms of T12/C13/A29/G30 base, and between C atoms of ethyl linker and C atoms of T14 base (shown in Table 3). These results predict that the distortion of DNA conformation in the Im-Pt(Testo)(II) + DNA adduct via the intercalative interaction mode is greater than that in the Pt(Testo)(II) + DNA adduct via the groove-face interaction mode.

### Binding of a non-testosterone-based platinum agent to DNA

Based on the previous experiments [26,27], a non-testosterone-based platinum complex displays little anticancer activity. We also performed MD simulation on the non-testosterone-based platinum agent, *cis*-[Pt(NH_3_)_2_(pyridine)Cl]^+^ (assigned as Pt(Py)(II)), in which a platinum(II) center is coordinated by one chlorine atom and three nitrogen atoms from two ammonias and only one pyridine, binding to DNA molecule (assigned as Pt(Py)(II) + DNA adduct). It can be found that there is hardly any change in DNA conformation and DNA groove parameters in the Pt(Py)(II) + DNA adduct compared with the undamaged B-DNA (Figure 4(b), (c), (d) and (e); see the blue lines with up-triangles and the black lines with squares for the Pt(Py)(II) + DNA and B-DNA models, respectively), except that the average deviation percentage of helical angle is 143.93% with respect to the undamaged B-DNA molecule (see Table 1). Simultaneously, there are no any destruction of hydrogen bond in the DNA molecule and formation of new hydrogen bond/hydrophobic interaction between the Pt(Py)(II) agent and DNA molecule in the Pt(Py)(II) + DNA adduct due to no testosterone ligand in the Pt(Py)(II) agent. These results suggest that the DNA conformation changes caused by the binding of the non-testosterone-based platinum agent are unconspicuous, which predicts that the non-testosterone-based Pt(Py)(II) agent is an inefficient anticancer agent reported by the previous experiment [27].

## Discussions

### Principal component analysis of major conformational dynamics

Principal component analysis was used to analyze the trajectories from the corresponding simulations to examine the dominant DNA dynamic motions in the studied Pt + DNA adducts. The first three principal components (PC1, PC2 and PC3) described about 80% of all the motion modes of dynamics for Pt(Testo)(II) + DNA, Im-Pt(Testo)(II) + DNA and Pt(Py)(II) + DNA adducts (see Table 4). It was found that the first three components of conformational motions roughly correspond to a superposition of bending, unwinding and twisting motions (see Additional file 4: Movie S1, Additional file 5: Movie S2 and Additional file 6: Movie S3, respectively, for the Im-Pt(Testo)(II) + DNA adduct). The overall average centroid structures of these adducts were analyzed and shown in Figure 3. Visual analyses of average DNA structures from the trajectories support the PCA dominant motions. It can be seen that the DNA conformations of Pt + DNA adducts with the groove-face and intercalative interaction modes including the bending, unwinding and twisting motions show obvious differences from the undamaged B-DNA conformation. For example, the percentages of occupancy times of PC1 and PC2 with the bending and unwinding motions, respectively, for both the Pt(Testo)(II) + DNA and Im-Pt(Testo)(II) + DNA adducts are larger than those for the Pt(Py)(II) + DNA adduct, which predicts that the platinum agents with the big-size testosterone ligand binding to DNA molecule via either groove-face interaction mode or intercalative interaction mode lead to greater bending and unwinding for DNA double helix than that without the testosterone ligand. Otherwise, the twisting motions for all three adducts make little contribution to the distortion of DNA conformation.

**Table 4:** Percentages of occupancy times of the first three principal components during the simulations of the studied Pt + DNA adducts

**Adducts**	**PC1**	**PC2**	**PC3**	**PCs**^**a**^
Pt(Testo)(II) + DNA	68.48	20.39	4.95	93.82
Im-Pt(Testo)(II) + DNA	57.34	19.65	8.34	85.33
Pt(Py)(II) + DNA	44.31	22.42	13.88	80.61

^a^: PCs represents sum of PC1, PC2 and PC3.

### DNA conformational dynamics in the Pt + DNA adducts with different interaction modes

**Figure 7:**
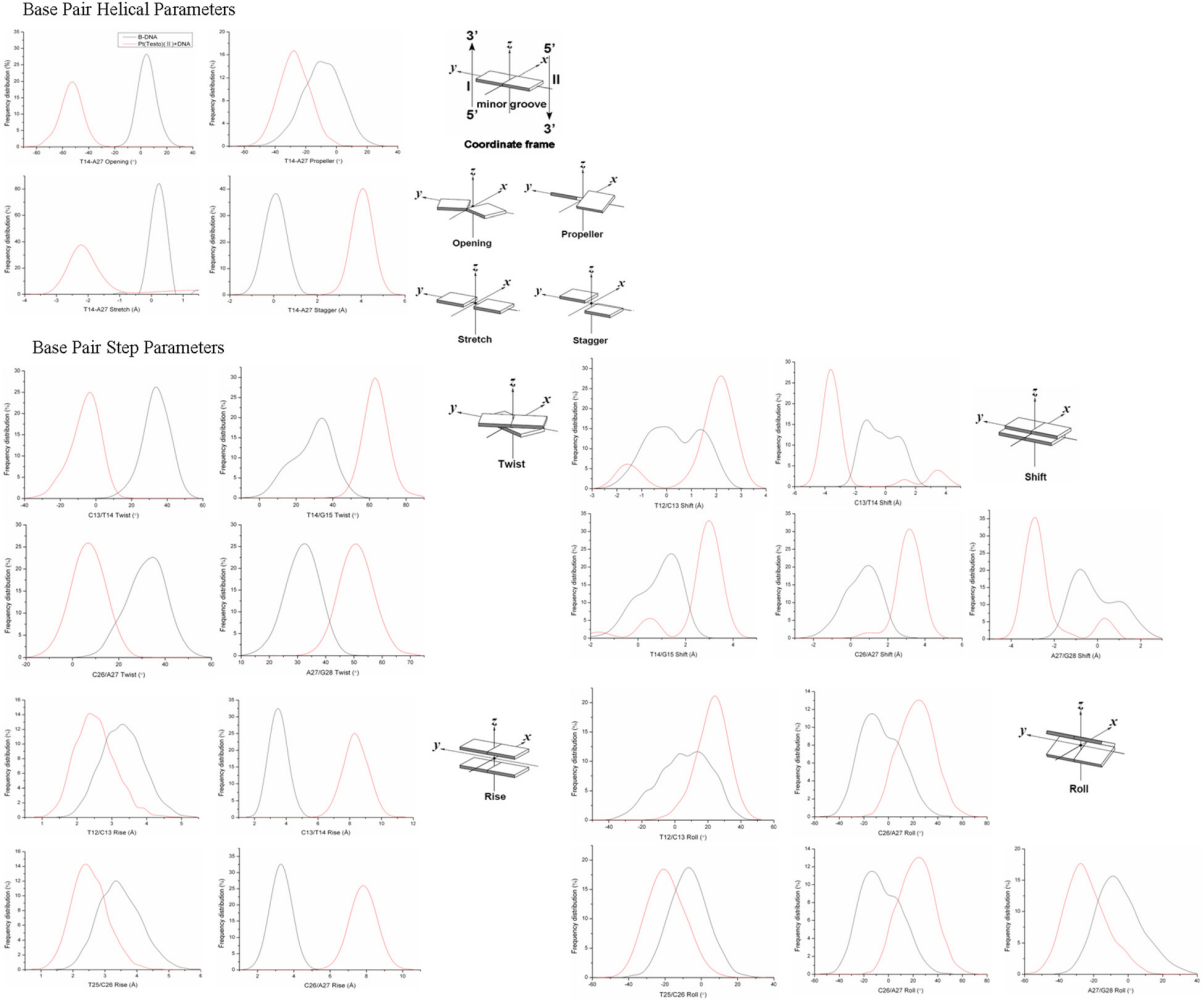
**Frequency distributions of DNA duplex base-pair helical/step parameters for Pt(Testo)(II) + DNA adduct and B-DNA.** Selected frequency distributions of representative DNA duplex base-pair helical/step parameters at the base pairs near the binding site and testosterone position for Pt(Testo)(II) + DNA adduct (red line) and B-DNA (black line).

The Pt(Testo)(II) and Im-Pt(Testo)(II) agents respectively via the groove-face and intercalative interaction modes locate at the major groove of DNA and disturb the DNA conformation greatly. The frequency distributions of DNA helical dynamics parameters at the base pairs near the binding site and testosterone position have been analyzed and shown in Figure 7 and Additional file 7: Figure S2 for the Pt(Testo)(II) + DNA and Im-Pt(Testo)(II) + DNA adducts, respectively, along with the distribution patterns of the undamaged B-DNA. Generally, the groove width and depth were measured mainly by the opening, twist and rise motions, and the shift motion for DNA bases, respectively, which has been defined by the Curves analysis [54-56].

In the Pt(Testo)(II) + DNA model, the opening parameter of DNA base pair helix near the testosterone position T14:A27 base pair is changed by ~ -50° away from the B-DNA molecule (see Figure 7); the deviations of twist angle values are ~ -35º, ~25º, ~-25º and ~20º for the C13 · T14, T14 · G15, C26 · A27 and A27 · G28 base pair steps with respect to ~ 36º for a base pair step of B-DNA molecule, in which the value of 25º is agreement with the unwinding angle of 21º determined by using gel mobility shift assays for the Pt(Testo)(II) agent in plasmid DNA solution [43]; moreover, the deviations of rise values are ~ -1.5Å, ~5Å and ~4.5Å for the T12 · C13, C13 · T14 and C26 · A27 base pair steps, respectively, with respect to ~ 3.5 Å for the B-DNA molecule (see Figure 7), which results in a widening of major groove by 1.55 Å and minor groove by 2.29 Å around the C11:G30 ~ G15:C26 base pairs (see Figure 4(a), (b) (red line) and (d) (red line)). The deviations of shift values are ~2Å, ~1.5Å, ~-2.5Å and ~ -2Å for the T12 · C13, T14 · G15, C26 · A27and A27 · G28 base pair steps, respectively, away from the B-DNA molecule (see Figure 7), which results in a shoaling of major groove by 3.96 Å and minor groove by 1.10 Å around the C11:G30 ~ G15:C26 base pairs (see Figure 4(a), (c) (red line) and (e) (red line)). In addition, the deviations of roll angle values are ~15°, ~-25º, ~40º and ~ -20° for the T12 · C13, C13 · T14, C26 · A27 and A27 · G28 base pair steps, respectively, away from the B-DNA molecule (see Figure 7), which contributes to the significant bend of DNA helix toward the major groove by 30.50°. Moreover, the conformational dynamics of DNA helical parameters in the vicinity of the testosterone position are associated with the pattern of hydrogen bond destruction and formation. For example, except of the opening parameter mentioned above, the propel, stagger, and stretch parameters for the DNA molecule in the Pt(Testo)(II) + DNA model near the testosterone position T14:A27 base pair are also changed by ~ -20°, ~4Å and ~ -2.5Å, respectively, away from the B-DNA molecule, which results in the destruction of hydrogen bonds at the base pair T14:A27. In addition, the DNA base motions of shift, twist at T14 · G15 and A27 · G28 base pair steps, and roll at A27 · G28 base pair step with the large deviations mentioned above (see Figure 7) are associated with the formations of new hydrogen bonds between the C-H groups of testosterone ligand and O6 atom of G28 base, and between the C-H groups of pyridyl ligand and O6 atom of G15 base with 20.82% and 13.78% occupancy times, respectively, for the Pt(Testo)(II) + DNA adduct (see Figure 6(a) and Table 2 ). These calculated results suggest that the DNA base dynamics motions caused by DNA-bound testosterone-based platinum agents induce DNA conformational distortion via the rearrangement of hydrogen bonds at the vicinity of testosterone position.

The deviation extents of DNA dynamics parameters in the Im-Pt(Testo)(II) + DNA model away from the B-DNA molecule are generally larger than those in the Pt(Testo)(II) + DNA model. For example, the deviations of twist angle values for the C26 · A27 base pair step in the Im-Pt(Testo)(II) + DNA model increases by 80% (from ~ -25º to ~ -45º) with respect to that in the Pt(Testo)(II) + DNA model. Especially, the deviation directions of some DNA dynamics parameters in the Im-Pt(Testo)(II) + DNA model are opposite to those in the Pt(Testo)(II) + DNA model. For example, the deviation direction of opening motion with ~65° at the T12:A29 base pair in the Im-Pt(Testo)(II) + DNA model is opposite to that with ~ -50° at the T14:A27 base pair in the Pt(Testo)(II) + DNA model, which suggests the difference of groove-face and intercalative interaction modes in the platinum + DNA adducts. These observations result from the intercalative interaction mode making the base pair step opening to the DNA major groove by a positive value, and oppositely the groove-face interaction mode making that opening to the minor groove by a negative value due to the testosterone ligand locating at the DNA major groove surface. These observations suggest that the different interaction modes of platinum agents with DNA molecules caused by the flexibility of platinum agents might greatly affect the DNA base dynamics motions and DNA conformational damage. Moreover, it can be seen in the present study that the intercalative interaction mode of platinum agent with DNA molecule might induce extensive DNA conformation distortion over the groove-face interaction mode in the platinum agent + DNA adducts.

## Conclusions

Molecular dynamics simulations and DNA dynamics analyses for a series of the Pt + DNA adducts were carried out to examine the distortions of DNA double-helical structure perturbed by the binding of testosterone-based platinum agent Pt(Testo)(II) (*cis*-[Pt(NH_3_)_2_(17α-pyridyl-3-ethyltestosterone)](II)), improved testosterone-based platinum agent Im-Pt(Testo)(II) (*cis*-[Pt(NH_3_)_2_(17α-pyridyl-3-ethynyltestosterone)](II)) and non-testosterone platinum agent Pt(Py)(II) (*cis*-[Pt(NH_3_)_2_(pyridine)](II)). It has been found that the rigid testosterone-based platinum agent Pt(Testo)(II) interacts with the DNA molecule via the groove-face interaction mode at the major groove of DNA; however, the improved flexible testosterone-based platinum agent Im-Pt(Testo)(II) interacts with DNA molecule via the intercalative interaction mode with the testosterone ligand inserting the middle of T12 and C13 bases. The distortion of DNA helical conformation caused by Im-Pt(Testo)(II) agent with intercalative interaction mode is larger than that caused by the Pt(Testo)(II) agent with groove-face interaction mode, which is supported by the formations of more hydrogen bonds and hydrophobic interactions at the platinum agent-DNA interface in the Im-Pt(Testo)(II) + DNA adduct. It can be found through the DNA dynamics analysis that the DNA base motions of opening, shift, rise, roll and twist away from the DNA groove caused by the binding of Pt(Testo)(II) and Im-Pt(Testo)(II) agents result in the widening and shoaling of DNA major/minor groove, and hydrogen bond destruction of DNA base pairs. Moreover, the non-testosterone-based platinum agent Pt(Py)(II) might cause insignificant change of DNA conformation due to the absence of testosterone ligand. Our simulation results might provide useful insights into understanding how a DNA conformation is affected by a testosterone-based platinum complex at the atomic level, and might be helpful for anticancer agent design.

## Competing interests

The authors declare that they have no competing interests.

## Authors’ contributions

SC carried out the computation and analysis of data, and drafted the manuscript. YW conceived this work and critically revised the manuscript. GC have made substantial contributions to the interpretation and evaluation of the results, and helped in construction of the manuscript. We also wish to thank the advice of our reviewers. All authors read and approved the manuscript.

## Supplementary Material

Additional file 1 **Table S1.** The optimized structure parameters by the B3LYP method for Pt(Testo)(II) agent along with the experiment data (bond Å and angle degree). Click here for file

Additional file 2 **Figure S1.** The chemical structure and numbering scheme of Pt(Testo)(II) agent. Click here for file

Additional file 3 Methodology. Click here for file

Additional file 4 Movie S1. Click here for file

Additional file 5 Movie S2. Click here for file

Additional file 6 Movie S3. Click here for file

Additional file 7 **Figure S2.** Frequency distributions of DNA duplex base-pair helical/step parameters for B-DNA and Im-Pt(Testo)(II) + DNA adduct. Selected frequency distributions of the representative DNA duplex base-pair helical/step parameters for at the base pairs near the binding site and testosterone position for B-DNA (black line) and Im-Pt(Testo)(II) + DNA adduct (Magenta line). Click here for file
