# Supplementary material for: Disturbance of DNA conformation by the binding of testosterone-based platinum drugs via groove-face and intercalative interactions: a molecular dynamics simulation study
Source: BMC Struct Biol. 2013 Mar 22;13:4. doi: 10.1186/1472-6807-13-4 (PMC3610147; doi:10.1186/1472-6807-13-4)
Supplement: Additional file 1 — Table S1. The optimized structure parameters by the B3LYP method for Pt(Testo)(II) agent along with the experiment data (bond Å and angle degree). [file 1472-6807-13-4-S1.doc]

**Table A1**. The optimized structure parameters by the B3LYP method for Pt(Testo)(Ⅱ) agent along with the experiment data (bond Å and angle degree)

|  | Calculated | Exiperment^a,b^ |  | Calculated | Exiperment^a,b^ |
| --- | --- | --- | --- | --- | --- |
| Pt(61)-Cl(66) | 2.333(9) | 2.300(7) | N(54)-Pt(61)-N(67) | 175.1(2) | 178.7(9) |
| Pt(61)-N(62) | 2.142(3) | 2.06(2) | N(67)-Pt(61)-N(62) | 94.7(9) | 90.2(9) |
| Pt(61)-N(67) | 2.107(0) | 2.04(2) | N(54)-Pt(61)-N(62) | 90.0(7) | 88.9(8) |
| Pt(61)-N(54) | 2.048(4) | 2.02(2) | N(67)-Pt(61)-Cl(66) | 85.1(4) | 89.1(7) |
| N(54)-C(52) | 1.347(5) | 1.35(3) | N(54)-Pt(61)-Cl(66) | 89.9(7) | 91.8(6) |
| N(54)-C(58) | 1.352(3) | 1.37(3) | N(62)-Pt(61)-Cl(66) | 179.2(6) | 178.8(7) |
| C(1)-C(2) | 1.543(3) | 1.536(5) | Pt(61)-N(54)-C(52) | 120.5(3) | 124 (1) |
| C(1)-C(6) | 1.553(0) | 1.533(4) | Pt(61)-N(54)-C(58) | 119.4(8) | 120(2) |
| C(1)-C(11) | 1.536(5) | 1.528(3) | C(1)-C(2)-C(3) | 104.4(7) | 103.5(3) |
| C(2)-C(3) | 1.554(2) | 1.542(5) | C(1)-C(6)-C(4) | 100.0(1) | 99.5(2) |
| C(3)-C(4) | 1.565(8) | 1.525(5) | C(1)-C(6)-C(7) | 113.3(1) | 113.7(2) |
| C(4)-O(5) | 1.425(2) | 1.436(5) | C(1)-C(6)-C(8) | 109.5(4) | 107.9(2) |
| C(4)-C(6) | 1.575(2) | 1.535(5) | C(1)-C(11)-C(10) | 107.6(2) | 109.0(2) |
| C(6)-C(7) | 1.545(7) | 1.536(5) | C(2)-C(1)-C(6) | 103.7(6) | 104.1(3) |
| C(6)-C(8) | 1.538(8) | 1.523(5) | C(2)-C(1)-C(11) | 120.4(6) | 119.6(2) |
| C(8)-C(9) | 1.545(3) | 1.538(5) | C(2)-C(3)-C(4) | 106.7(4) | 106.6(3) |

^a^ Taken from Ref [1]

^b^ Taken from Ref [2]

**References**

1. Hollis LS, Lippard SJ: **Mononuclear complexes of cis-diammineplatinum(II) and -(IV) with .alpha.-pyridone. Structures of cis-[Pt(NH3)2(C5H4NOH)2]Cl2, mer-[Pt(NH3)2(C5H4NO)Cl3], and cis-[Pt(NH3)2(C5H4NOH)Cl](NO3)**. *Inorganic Chemistry* 1983, **22**:2708-2713.

2. Roberts PJ, Pettersen RC, Sheldrick GM, Isaacs NW, Kennard O: **Crystal and molecular structure of 17β-hydroxyandrost-4-en-3-one (testosterone)**. *J Chem Soc, Perkin Trans 2* 1973:1978-1984.
