# Supplementary figures and images for: Disturbance of DNA conformation by the binding of testosterone-based platinum drugs via groove-face and intercalative interactions: a molecular dynamics simulation study
Source: BMC Struct Biol. 2013 Mar 22;13:4. doi: 10.1186/1472-6807-13-4 (PMC3610147; doi:10.1186/1472-6807-13-4)

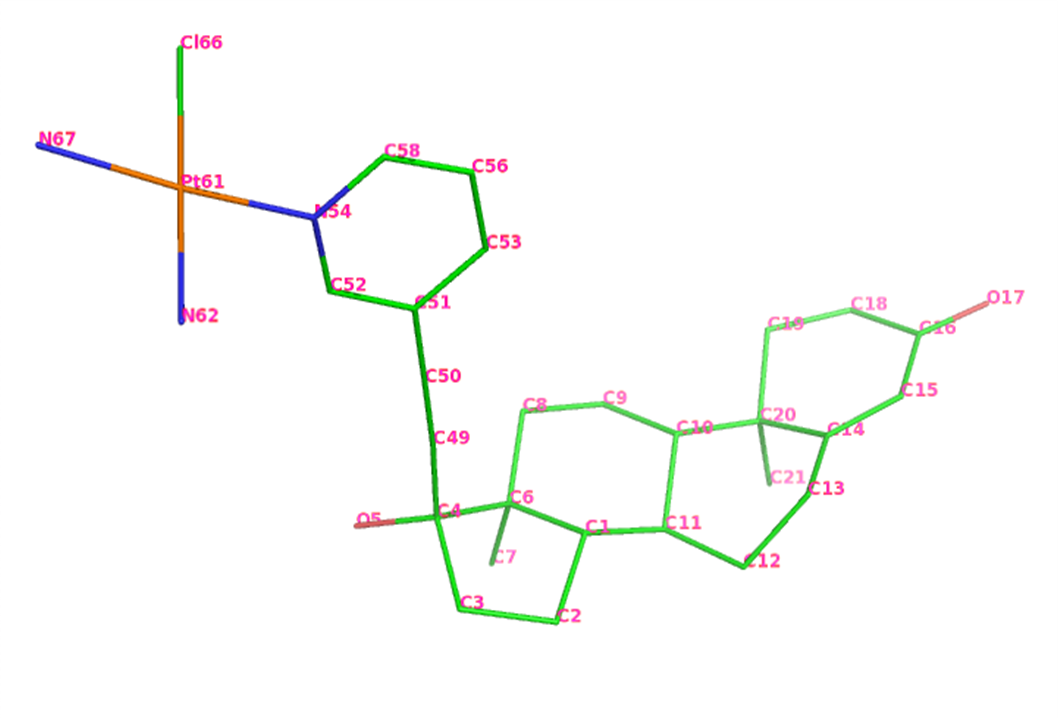

Supplement: Additional file 2 — Figure S1. The chemical structure and numbering scheme of Pt(Testo)(II) agent. [file 1472-6807-13-4-S2.doc]

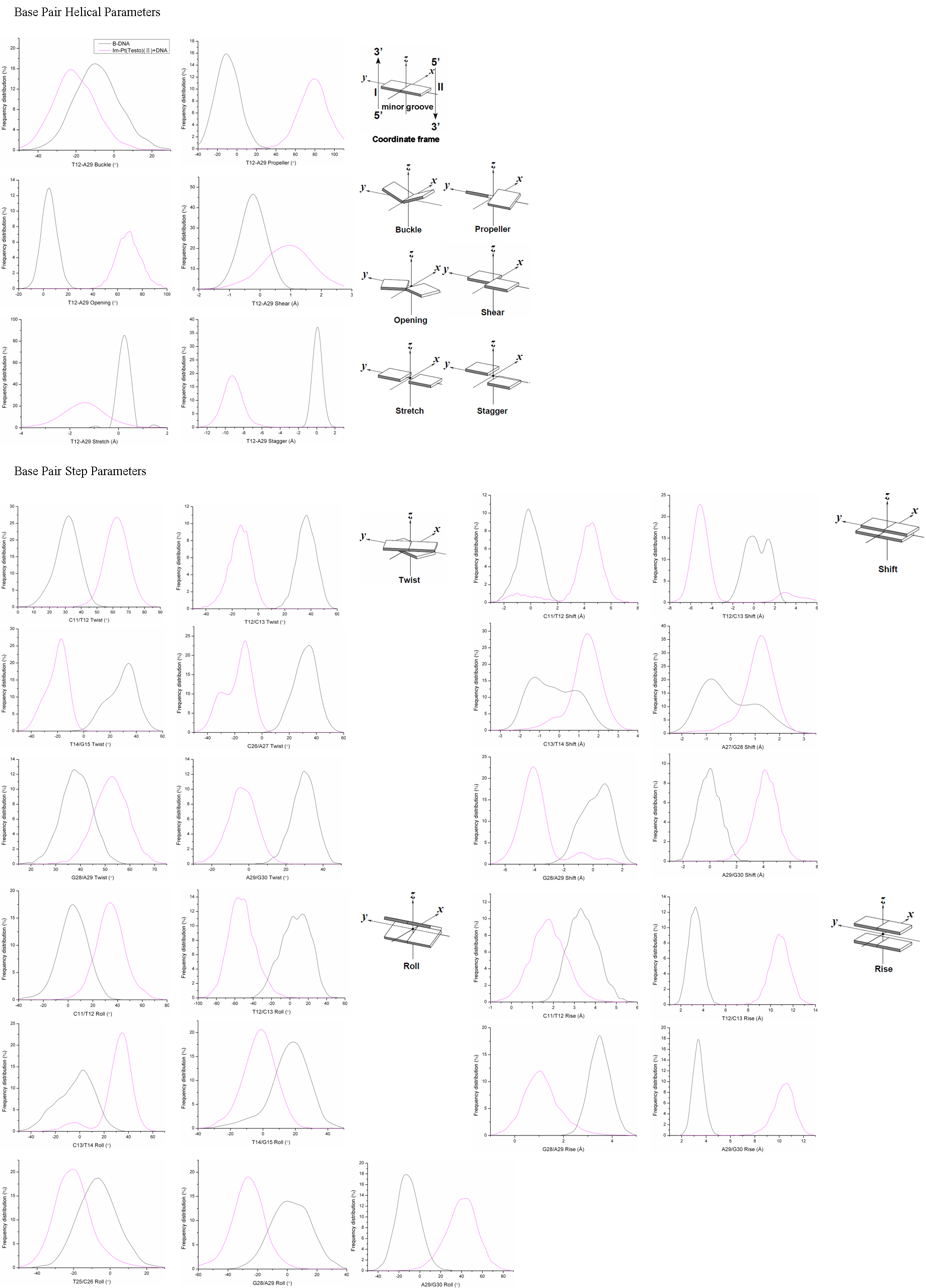

Supplement: Additional file 7 — Figure S2. Frequency distributions of DNA duplex base-pair helical/step parameters for B-DNA and Im-Pt(Testo)(II) + DNA adduct. Selected frequency distributions of the representative DNA duplex base-pair helical/step parameters for at the base pairs near the binding site and testosterone position for B-DNA (black line) and Im-Pt(Testo)(II) + DNA adduct (Magenta line). [file 1472-6807-13-4-S7.doc]
