# Supplementary material for: Disturbance of DNA conformation by the binding of testosterone-based platinum drugs via groove-face and intercalative interactions: a molecular dynamics simulation study
Source: BMC Struct Biol. 2013 Mar 22;13:4. doi: 10.1186/1472-6807-13-4 (PMC3610147; doi:10.1186/1472-6807-13-4)
Supplement: Additional file 3 — Methodology. [file 1472-6807-13-4-S3.doc]

**Additional Materials**

**Molecular dynamics simulation protocols**

All MD simulations were carried out using the AMBER9 package with a classical AMBER parm99 together with the parmbsc0 refinement and gaff force field parameters. The protocol for all MD simulations is as follows: (1) All systems were submitted to energy minimization to remove unfavorable contacts. Four cycles of minimizations were performed with 2500 steps in each minimization with harmonic restraints ranging from 100 kcal mol^-1^Å^-2^, 25 kcal mol^-1^Å^-2^ to 10 kcal mol^-1^Å^-2^ on DNA and the platinum compound positions. Finally, 5000 steps of unrestrained minimization were carried out before the heating process. The cutoff distance used for non-bonded interactions was 10Å. The SHAKE algorithm was used to restrain the bonds containing hydrogen. (2) Each energy-minimized structure was heated over 120 ps from 0 to 300K (with a temperature coupling of 0.2 ps) in a constant volume, while the positions of DNA and the platinum compound were restrained with a small value of 10 kcal mol^-1^Å^-2^. The constant volume was maintained during the processes. (3) The unrestrained equilibration of 200 ps with constant pressure and constant temperature conditions was carried out before a trajectory was generated for a further production simulation. The temperature and pressure were allowed to fluctuate around 300K and 1 bar respectively with a corresponding coupling of 0.2 ps. For each simulation, an integration step of 2 fs was used. (4) Production runs of 50 ns were carried out by following the same protocol. A time point of 200 ps after thermal equilibration in each simulation was selected as a starting point for data collection. During the production run, 25000 structures from each simulation were saved for post-processing by uniformly sampling the trajectory. (5) Finally, simulated annealing was performed for each system by allowing the system to cool from 300 to 0K during the course of 1 ns simulation.

**Principal component analysis**

Principle component analysis (PCA) was used to identify the dominant motions observed during a simulation by visual inspection by calculating the eigenvectors from the covariance matrix of a simulation and then filtering the trajectories along each of the different eigenvectors. As PCA reduces the dimensionality of the trajectory data, using only the first several principal components we can extract important dynamical features describing collective and overall motions of the system . The Pt-DNA adduct systems reached equilibrium after 20 ns, thus final 30 ns of equilibrated MD simulations of each of the 15000 trajectories of Pt-DNA adducts were subject to PCA analysis. The first three principal components described >80% of the essential modes of dynamics for the platinum adducted DNA complexes. The dynamics of the first three principal components were visualized using the *Discovery Studio* molecular graphics tool.

**DNA groove parameter analyses of trajectories**

The PTRAJ module of the AMBER9 program was used to extract production conformations. These extracted snapshots were saved in the Protein Data Bank (PDB) format. Each nucleotide type was converted from the AMBER format to PDB format, and the resulting snapshots were submitted to the CURVES program . The following CURVES parameters were extracted, i.e. global base-base parameters: shear, stretch, stagger, buckle, propeller and opening; global inter base-pair parameters: shift, slide, rise, tilt, roll and twist. Percentage occupancy distributions of the DNA helical parameters were calculated by normalizing the frequency distributions to 100%.

The overall bend, tilt and roll angles of DNA for the average structures of these adducts were calculated from the CURVES output using MadBend (<http://www.biomath.nyu.edu/index/software/Madbend/index.html>) developed by Strahs and Schlick. This method evaluates the DNA curvature by summing the projected components of local base-pair step tilt and roll angles after adjusting the helical twist. Bends in the helical axis defined by a negative roll angle indicate bending toward the minor groove, while bends defined by a positive roll angle correspond to bending toward the major groove .

**References**

1. Case DA, Darden TA, Cheatham ITE, Simmerling CL, Wang JM, Duke RE, Luo R, Merz KM, Pearlman DA, Crowley M: **AMBER9**. *University of California, San Francisco* 2006.

2. Duan Y, Wu C, Chowdhury S, Lee MC, Xiong G, Zhang W, Yang R, Cieplak P, Luo R, Lee T: **A point-charge force field for molecular mechanics simulations of proteins based on condensed-phase quantum mechanical calculations**. *Journal of computational chemistry* 2003, **24**:1999-2012.

3. Lee MC, Duan Y: **Distinguish protein decoys by using a scoring function based on a new AMBER force field, short molecular dynamics simulations, and the generalized born solvent model**. *Proteins: Structure, Function, and Bioinformatics* 2004, **55**:620-634.

4. Yao S, Plastaras JP, Marzilli LG: **A molecular mechanics AMBER-type force field for modeling platinum complexes of guanine derivatives**. *Inorganic chemistry* 1994, **33**:6061-6077.

5. Pérez A, Marchán I, Svozil D, Sponer J, Cheatham III TE, Laughton CA, Orozco M: **Refinement of the AMBER force field for nucleic acids: improving the description of [alpha]/[gamma] conformers**. *Biophysical journal* 2007, **92**:3817-3829.

6. Wang J, Wolf RM, Caldwell JW, Kollman PA, Case DA: **Development and testing of a general amber force field**. *Journal of computational chemistry* 2004, **25**:1157-1174.

7. Miyamoto S, Kollman PA: **Settle: an analytical version of the SHAKE and RATTLE algorithm for rigid water models**. *Journal of computational chemistry* 1992, **13**:952-962.

8. Amadei A, Linssen A, Berendsen HJC: **Essential dynamics of proteins**. *Proteins: Structure, Function, and Bioinformatics* 1993, **17**:412-425.

9. Yamaguchi H, van Aalten DMF, Pinak M, Furukawa A, Osman R: **Essential dynamics of DNA containing a cis. syn cyclobutane thymine dimer lesion**. *Nucleic acids research* 1998, **26**:1939-1946.

10. Teeter MM, Case DA: **Harmonic and quasiharmonic descriptions of crambin**. *Journal of Physical Chemistry* 1990, **94**:8091-8097.

11. Van Aalten D, Findlay J, Amadei A, Berendsen H: **Essential dynamics of the cellular retinol-binding protein evidence for ligand-induced conformational changes**. *Protein engineering* 1995, **8**:1129-1135.

12. Lavery R, Sklenar H: **The definition of generalized helicoidal parameters and of axis curvature for irregular nucleic acids**. *Journal of biomolecular structure & dynamics* 1988, **6**:63.

13. Strahs D, Schlick T: **A-tract bending: insights into experimental structures by computational models**. *Journal of molecular biology* 2000, **301**:643-663.
